# Diverse Facets of Sphingolipid Involvement in Bacterial Infections

**DOI:** 10.3389/fcell.2019.00203

**Published:** 2019-09-19

**Authors:** Tobias C. Kunz, Vera Kozjak-Pavlovic

**Affiliations:** Department of Microbiology, Biocenter, University of Würzburg, Würzburg, Germany

**Keywords:** infection, pathogenic bacteria, sphingolipids, ceramide, autophagy

## Abstract

Sphingolipids are constituents of the cell membrane that perform various tasks as structural elements and signaling molecules, in addition to regulating many important cellular processes, such as apoptosis and autophagy. In recent years, it has become increasingly clear that sphingolipids and sphingolipid signaling play a vital role in infection processes. In many cases the attachment and uptake of pathogenic bacteria, as well as bacterial development and survival within the host cell depend on sphingolipids. In addition, sphingolipids can serve as antimicrobials, inhibiting bacterial growth and formation of biofilms. This review will give an overview of our current information about these various aspects of sphingolipid involvement in bacterial infections.

## Introduction

**FIGURE 1:**
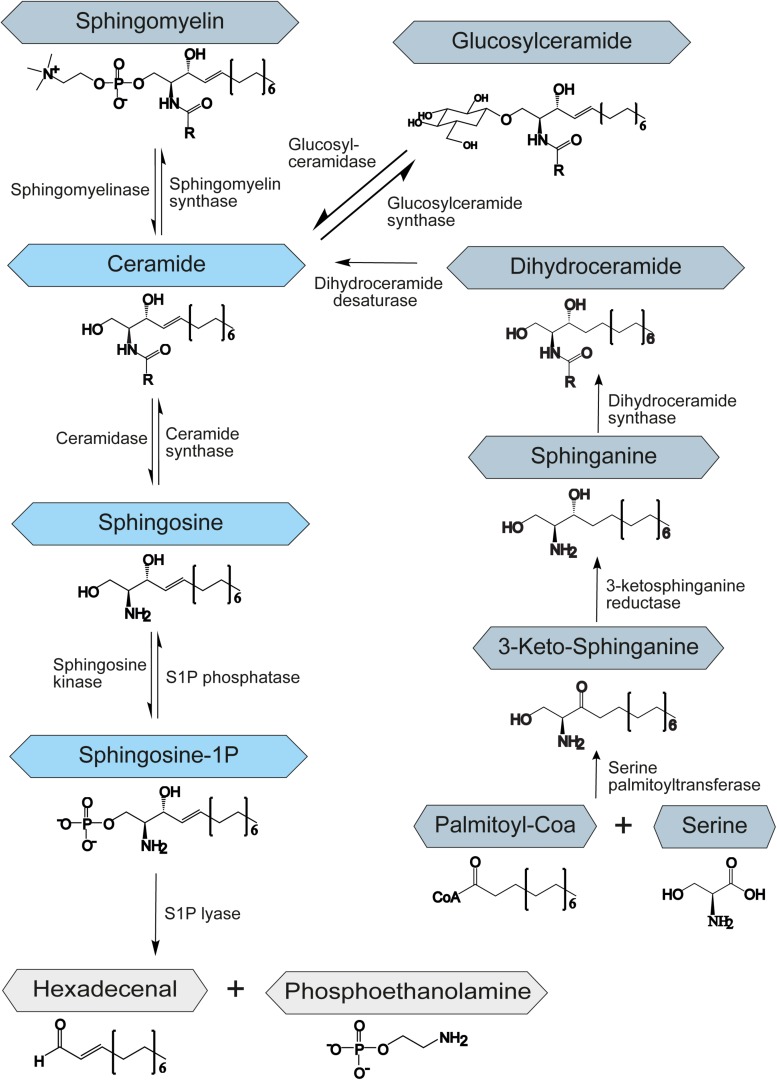
Schematic representation of the sphingolipid metabolism. S1P – sphingosine-1-phosphate. The central role of the sphingolipid metabolism plays ceramide. It is synthesized *de novo* from serine and palmitoyl-CoA, by hydrolysis of sphingomyelin or through the salvage pathway by recovery of sphingosine from complex sphingolipids. Degradation of sphingolipids occurs through the degradation of S1P to hexadecenal and phosphoethanolamine by an enzyme called S1P-lyase.

Sphingolipids belong to a class of lipids defined by their amino-alcohol backbone. They were considered merely to be ubiquitous components of the eukaryotic cell membrane, shown to play a critical role in the formation of membrane microdomains called lipid rafts that are important for cell signaling (Simons and Ikonen, 1997). However, in the past decades, it has been revealed that many sphingolipids are bioactive lipids that regulate a large subset of cellular functions, such as apoptosis or autophagy (Cuvillier et al., 1996; Harvald et al., 2015). Although sphingolipids vary greatly in their structure and function, their synthesis and degradation are mediated by common synthetic and catabolic pathways. Sphingolipids can be synthesized *de novo*, by the hydrolysis of sphingomyelin, or through the salvage pathway, by recovery of sphingosine from complex sphingolipids (Figure 1). In all cases the result is the synthesis of ceramide, which represents the starting point for the creation of complex sphingolipids and thus is involved in many regulating processes within the cell. The metabolism of sphingolipids has been extensively reviewed by Gault et al. (2010). Ceramide itself regulates growth and development and promotes cell survival and division (Mencarelli and Martinez-Martinez, 2013).

The *de novo* biosynthesis of ceramide starts at the endoplasmic reticulum (ER) with an enzyme called serine palmitoyltransferase. This enzyme catalyzes the condensation of serine and fatty acid-CoA to 3-ketosphinganine. Afterward, 3-ketosphinganine gets reduced by 3-ketosphinganine reductase and then is processed by dihydroceramide synthase to dihydroceramide. In the final step of the *de novo* ceramide biosynthesis, a dihydroceramide desaturase introduces a double bond to create ceramide (Perry, 2002; Menaldino et al., 2003; Figure 1).

Constitutive degradation of sphingolipids and glycosphingolipids takes place in the late endosomes and lysosomes at acidic pH to form sphingosine (Riboni et al., 1997; Kolter and Sandhoff, 2005). The oligosaccharide chains of glycosphingolipids are stepwise removed by the release of monosaccharide units through exohydrolases. In the salvage pathway, long-chain sphingoid bases are broken down to sphingosine, which is reacylated to form ceramide by an enzyme called ceramide synthase. Thus, ceramide synthase family members probably trap free sphingosine released from the lysosome at the surface of the ER or in ER-associated membranes. The salvage pathway is estimated to contribute to 50–90% of sphingolipid biosynthesis (Gillard et al., 1998; Tettamanti et al., 2003).

During the generation of ceramide from sphingosine, ceramide synthases add different fatty acyl chains at the C2-amino group of the sphingosine backbone, resulting in numerous and diverse sphingolipids. Variations in the chain length of ceramide acyl chains are linked to potentially altered membrane bilayer dynamics or differential signaling properties by recruitment of different binding partners. This topic has previously been reviewed by Grösch et al. (2012). However, the effect of different length of acyl chains on ceramide properties and function is poorly understood and needs further investigation.

Ceramide exerts a specific function in mitochondria, where the increase in ceramide has been linked to the induction of apoptosis. Ceramide pool in mitochondria seems to be regulated by the localized activity of ceramide synthase, sphingomyelinases, and neutral ceramidase. However, another source of mitochondrial ceramide could be the ER, due to the proximity and close interaction of these two organelles (Hernández-Corbacho et al., 2017).

Apart from ceramide, sphingosine-1-phosphate (S1P) has been shown to be a potent signaling molecule. It is linked to the regulation of mitochondrial function (Bajwa et al., 2015), gene expression (Davaille et al., 2000), and ER stress (Lépine et al., 2011). Moreover, it was implicated in the regulation of important processes such as apoptosis, autophagy, and cell proliferation (Harvald et al., 2015). S1P is synthesized by the sphingosine kinase-1 and -2 (SPHK1/2) by phosphorylation of sphingosine and degraded by the S1P phosphatase (SGPP) or lyase (SGPL1) to sphingosine or hexadecenal and phosphoethanolamine, respectively (Figure 1). While SPHK1 is mainly associated with cell survival (Sarkar et al., 2005), SPHK2 has been shown to influence mitochondrial function and homeostasis. It has also been involved in regulation of histone deacetylases and thereby in suppression of cell growth and promotion of apoptosis (Liu et al., 2003). SGPP and SGPL1 ensure balanced levels of S1P and other sphingolipid intermediates that may control cell growth and death. The upregulation of SGPL1 results in an accumulation of hexadecenal, which was shown to be cytotoxic (James and Zoeller, 1997).

In the last years, sphingolipids have been revealed as key players in infection processes. Even though most pathogenic bacteria do not produce their own sphingolipids, they are capable of utilizing or degrading host sphingolipids to promote their virulence. This review will summarize some of our current knowledge about involvement of sphingolipids in bacterial infection, starting from the interaction with pathogenic bacteria on the surface of the cell, including the uptake of bacteria, immune response, survival and propagation of intracellular bacteria, and ending with several remarks on the bactericidal effects of sphingolipids.

## Bacterial Entry - Role of Sphingolipids in Bacterial Adhesion and Uptake

**FIGURE 2:**
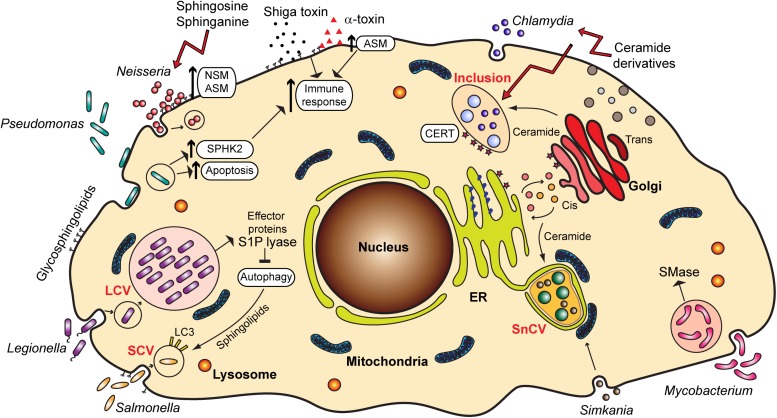
Overview of the various bacterial pathogens and their interaction with sphingolipids and sphingolipid signaling pathways. ER, endoplasmic reticulum; LCV, *Legionella*-containing vacuole; SCV, *Salmonella*-containing vacuole; SnCV, *Simkania negevensis*-containing vacuole; ASM, acid sphingomyelinase; NSM, neutral sphingomyelinase; SPHK2, sphingosine kinase 2; S1P, sphingosine-1-phosphate; SMase, sphingomyelinase; CERT, ceramide transfer protein; LC3, Microtubule-associated protein 1A/1B-light chain 3.

Glycosphingolipids frequently serve as receptors for *Escherichia coli* and other bacteria such as *Pseudomonas aeruginosa*, *Bordetella pertussis*, *Mycoplasma pneumoniae*, and *Helicobacter pylori* [reviewed in Hanada (2005)]. A prominent example is GM1 ganglioside, which serves as a receptor for cholera toxin (Holmgren et al., 1975). Also, ceramide-enriched lipid rafts acting as binding platforms, as well as sphingolipid signaling, such as through the activation of acid sphingomyelinase (ASM) (Simonis and Schubert-Unkmeir, 2018), often mediate the entry of bacterial pathogens into host cells, which is a step important for infection and establishment of bacteria in an intracellular niche (Figure 2).

For pathogenic *Neisseria*, sphingolipids play an important role in the adhesion and invasion into the host cell. *Neisseria meningitidis*, a causative agent of meningitis and meningococcal sepsis, similar to *Haemophilus influenzae* binds to specific glycosphingolipids on the host cell surface, which can be found on human granulocytes and oropharyngeal epithelium, the preferential habitat for these two pathogens (Hugosson et al., 1998). *Neisseria* express highly variable lipooligosaccharides (LOS) on their surface, molecular mimics of glycosphingolipids found on human cells. LOS undergo phase variation, which is important for immune evasion, as well as adherence and invasion (Harvey et al., 2001).

Several reports emphasize the role of sphingomyelinases in the invasion of pathogenic *Neisseria*. The activation of ASM by *N. gonorrhoeae*, a bacterium causing sexually transmitted disease gonorrhea, is important for the entry of these bacteria into non-phagocytic cells (Grassmé et al., 1997), which occurs through the Opa-mediated interaction of bacteria with carcinoembryonic antigen-related cell adhesion molecule (CEACAM) receptors (Hauck et al., 2000). Neutral sphingomyelinase (NSM) also plays a role in the uptake of *N. gonorrhoeae* (Faulstich et al., 2015), but by a different mechanism, the so-called PorB_*IA*_-mediated low phosphate-dependent invasion (Kühlewein et al., 2006). *N. meningitidis* likewise causes the activation of ASM and ceramide release that are essential for the internalization of meningococci into brain endothelial cells, which is connected to the expression of the outer membrane protein OpcA and binding to cell surface heparan sulfate proteoglycans (HSPGs) (Simonis et al., 2014). Recently, a role of meningococcal pilus in the translocation of ASM to the surface of infected cells has been described (Peters et al., 2019). In all cases, invasion of the host cell contributes to immune evasion and spreading of these pathogenic bacteria from the site of the initial contact to other tissues.

Uptake of bacteria through activation of sphingomyelinases as seen for *Neisseria* is not always beneficial for the pathogen but represents a host defense mechanism, as well. For example, *P. aeruginosa*, associated with serious hospital-acquired and opportunistic infections, activates host ASM, leading to generation of plasma membrane ceramide-enriched platforms that mediate the internalization of bacteria, apoptosis induction and cytokine response (Grassme et al., 2003). In macrophages, as well as in neutrophils, infection with *P. aeruginosa* leads to cell death, which is important for the clearing of infection. In both cases the role of sphingomyelinases in this process has been proposed. For alveolar macrophages, after the activation of the ASM upon infection, the formation of ceramide-enriched platforms serves to amplify ASM-mediated redox signaling, which eventually leads to macrophage apoptosis (Zhang et al., 2008). In the case of neutrophils, the apoptosis is mediated through pyocyanin, a pigment and toxin released by *P. aeruginosa*, and through mitochondrial ASM (Managò et al., 2015). However, *P. aeruginosa* can possibly counteract the increase in ceramide through secretion of hemolytic phospholipase C that can synthesize sphingomyelin from ceramide, and alkaline ceramidase, which can break down ceramide to avoid generation of ceramide-enriched platforms and uptake, and increase hemolysis (Okino et al., 1998; Okino and Ito, 2007).

Not only ceramide, but sphingosine and S1P play a part in lung inflammatory injury caused by *P. aeruginosa*. SPHK2 is phosphorylated upon *P. aeruginosa* infection, which increases its nuclear localization, leading to higher levels of S1P and increased histone acetylation. This as a consequence has an enhanced expression of pro-inflammatory genes and secretion of pro-inflammatory cytokines, interleukin-6, and tumor necrosis factor-α (Ebenezer et al., 2019). Lastly, surface sphingolipids are also involved in infection with *P. aeruginosa*. The bacterial pilin binds to asialo GM1, but not to sialylated gangliosides. Considering that the surface of epithelial cells from cystic fibrosis patients shows differences in sialylation of glycolipids, this might enhance susceptibility of cystic fibrosis patients to infections with *P. aeruginosa* (Saiman and Prince, 1993).

Sphingolipids seem also to play a specific role in the internalization of *Mycobacteria*, among which are the causative agents of tuberculosis and leprosy, by macrophages, as shown for *Mycobacterium smegmatis*. For this bacterium, the metabolic depletion of sphingolipids in J774a.1 macrophages led to a decrease in entry of bacteria into the host cells (Viswanathan et al., 2018).

Finally, the pathogenicity of bacteria is often mediated by the binding of bacterial toxins to gangliosides and glycosphingolipids, but this aspect of the role of sphingolipids in bacterial infections will not be reviewed here in detail. Prominent examples are Shiga toxin from *Shigella dysenteriae* [reviewed in Kavaliauskiene et al. (2017)] and *Staphylococcus aureus* and its α-toxin. α-toxin has been reported to activate ASM, which leads to changes in permeability and lung edema (Becker et al., 2017). Ceramide increase upon *S. aureus* infection also stimulates pro-inflammatory signaling through a release of cathepsin B and D from lysosomes (Ma et al., 2017; Figure 2). Therefore, the inflammation, edema and lung tissue damage upon *S. aureus* infection seem to be induced by the binding of α-toxin and activation of ASM.

Inhibition of sphingomyelinases as a means of controlling inflammation and spread of infection might be consequently beneficial for *S. aureus* infection (Peng et al., 2015), or infection with pathogenic *Neisseria*, but is detrimental in case of infection with *P. aeruginosa* (Grassme et al., 2003). It is therefore of great importance to understand the mechanisms underlying infections with different pathogenic bacteria, so that this knowledge could be translated to appropriate treatments.

## Once Inside - Role of Sphingolipids in Bacterial Reproduction and Survival

Uptake of bacteria into cells can be a mechanism for bacterial killing, through formation of phagolysosomes or induction of autophagy. However, bacteria can avoid death by escaping into the cytosol, preventing fusion of phagosomes and lysosomes, blocking autophagy, or, in some cases, surviving within the lysosomal environment. Certain bacteria also reside in non-lysosomal compartments inside the cell where they multiply, avoiding recognition and immune response. Sphingolipids have important function in many of these strategies for the survival of pathogenic bacteria within the host cell.

### Sphingolipids in Bacterial Development and Reproduction

Obligate intracellular bacteria heavily depend on the host cell for the procurement of the metabolites required for their development. Sphingolipids represent important building blocks for the membranes of compartments in which these bacteria reside and multiply (Figure 2).

*Chlamydia trachomatis*, a causative agent of trachoma and sexually transmitted disease, is characterized by a biphasic life cycle, in which a reproductive part is spent inside the host cell, within a vesicular compartment known as an inclusion. *Chlamydia* directly obtain ceramide from the host cell Golgi and incorporate it into the inclusion membrane (Hackstadt et al., 1995; Figure 2). This is a process essential for the pathogen survival, in which chlamydial inclusion fuses with *trans-*Golgi network-derived secretory vesicles (van Ooij et al., 2000) in an Akt and Rab14-mediated way (Capmany and Damiani, 2010; Capmany et al., 2019). *C. trachomatis* has also been reported to cause the fragmentation of Golgi and to induce formation of mini stacks in the vicinity of the inclusion membrane, which supports lipid acquisition from the host (Heuer et al., 2009). Interestingly, it is possible that *C. trachomatis* establishes a sphingomyelin biosynthetic factory at or near the inclusion with the help of host cell proteins. Moreover, Elwell et al. (2011) showed that *C. trachomatis* co-opts Golgi-specific Brefeldin A resistance guanine nucleotide exchange factor 1 (GBF1), a protein important for the assembly and maintenance of the Golgi stack, for sphingomyelin acquisition contributing to the growth and stability of the inclusion. For the replication, however, *C. trachomatis* recruits ceramide transfer protein (CERT), vesicle-associated membrane protein-associated protein A (VAP-A), and sphingomyelin synthases, SMS1 and SMS2 to the inclusion membrane, therewith obtaining ceramide and converting it into sphingomyelin close to the inclusion (Elwell et al., 2011). Recruitment of CERT occurs with the help of chlamydial effector protein IncD present in the inclusion membrane (Agaisse and Derre, 2014). Other reports implicate additionally the Src family tyrosine kinase Fyn (Mital and Hackstadt, 2011), as well as *trans-*Golgi SNARE protein syntaxin 6 (Moore et al., 2011) in sphingolipid trafficking to chlamydial inclusion.

The described acquisition of sphingolipids from the host cells seems to be a common occurrence among chlamydia. Both *Chlamydia pneumoniae* and *Chlamydia psittaci* follow similar mechanisms, involving exocytic vesicles or CERT protein (Wolf and Hackstadt, 2001; Koch-Edelmann et al., 2017). In addition, a chlamydia-like microorganism *Simkania negevensis* has been likewise reported to obtain ceramide from the host cell, a process most likely dependent on the retrograde transport (Herweg et al., 2016).

*Mycobacterium tuberculosis*, although not strictly intracellular, uses host macrophages and dendritic cells for replication. During this process, the mycobacteria have been shown to depend on sphingomyelin. *M. tuberculosis* express the protein Rv0888, which exhibits a sphingomyelinase activity and cleaves host sphingomyelin into ceramide and phosphorylcholine. These compounds are used by bacteria as the sources of carbon, nitrogen and phosphorus (Speer et al., 2015). However, the purpose of *M. tuberculosis*-produced sphingomyelinases might be not only to provide a source of nutrients, but to modulate sphingolipid signaling and cell death induction, thereby controlling the immune response to this pathogen (Castro-Garza et al., 2016).

### Sphingolipid Importance for the Survival of Intracellular Bacteria

Autophagy and apoptosis play a crucial role in controlling infection with various bacteria and viruses, representing an important part of innate immunity, but also being manipulated occasionally by pathogens for the purpose of survival or replication (Rudel et al., 2010; Siqueira et al., 2018). Sphingolipids were shown to be involved in the regulation of both apoptosis and autophagy, with ceramide being associated with cell death and S1P promoting cell survival (Young et al., 2013). Increase in intracellular levels of ceramide or treatment with sphingomyelinase has been reported to induce apoptosis in HL-60 or U937 cells, an effect which could be prevented by exposure of the cells to S1P (Cuvillier et al., 1996). For this, the term “sphingolipid rheostat” has been introduced to describe regulation of cell fate through the interconversion between ceramide and S1P. However, a multitude of later studies has shown a great complexity of the signaling mechanisms by which these sphingolipid metabolites influence cell death (Newton et al., 2015). As bacteria often modulate apoptosis to accommodate the host cell to their own needs (Rudel et al., 2010) it is possible that this modulation partially takes place through sphingolipid signaling, and that mitochondrial sphingolipids play a special role in this process. This direction is certainly worth exploring in the future, especially for intracellular bacteria.

In addition to apoptosis, autophagy and the associated cell death have also been subject to regulation by “sphingolipid rheostat” (Taniguchi et al., 2012; Young et al., 2013). Autophagy is a highly conserved catabolic process through which unnecessary or damaged components are degraded to maintain cellular homeostasis (Young et al., 2013; Young and Wang, 2018). In brief: a cargo is engulfed by a membrane forming the so-called autophagosome. Mature autophagosomes can then fuse with lysosomes, leading to the degradation of the cargo (Miller and Celli, 2016). It has been shown that S1P upregulates autophagy, therefore promoting cell survival, although several reports indicated that S1P can also act as an inhibitor of autophagy through activation of the mammalian target of rapamycin (mTOR) (Harvald et al., 2015). Ceramide induces autophagy and mitophagy, could be important for fusion of autophagosomes with lysosomes, and is involved in induction of autophagic cell death. In addition, the “many ceramides” hypothesis implies that the function of ceramide depends on the chain length, adding a level of complexity to the regulation of autophagy through sphingolipids (Young et al., 2013; Harvald et al., 2015). Autophagy can be further subdivided in selective and non-selective. Non-selective autophagy describes the degradation of a random portion of a cytosol to provide nutrients during starvation or to degrade long-lived proteins. Selective autophagy targets specific cellular compartments. This includes utilizing the autophagy machinery for the removal of mitochondria (mitophagy) or the clearance of intracellular pathogens (xenophagy) (Deretic et al., 2013; Huang and Brumell, 2014). Hence, evading or regulating autophagy is important for the pathogen survival.

One well-studied pathogen regulating the autophagic machinery is *Legionella pneumophila*, a gram-negative bacterium naturally replicating in protists in aquatic environments. It is the causative agent of the Legionnaires‘ disease (Newton et al., 2010). As many signaling pathways are conserved in human macrophages and protists, *L. pneumophila* are capable of invading human cells, where they replicate in vacuoles, the so-called *Legionella-*containing vacuole (LCV). Inside the cell, *L. pneumophila* secretes over 300 effector proteins interfering with a broad range of cellular pathways. It has been shown that many of these effector proteins have a structure similar to eukaryotic proteins that are never or rarely found in prokaryotic genomes. It is hypothesized that *L. pneumophila* acquired these proteins through horizontal gene transfer from its host (Cazalet et al., 2004; Gomez-Valero and Buchrieser, 2013). Among those proteins, three proteins share similarities to eukaryotic proteins of the sphingolipid pathway: Lpp2641 (putative sphingomyelinase), Lpp2295 (putative sphingosine kinase) and Lpp2128 or *Lp*SPL (S1P lyase) (Rolando et al., 2016a). The latter, being a S1P lyase, leads to the degradation of S1P, which is a critical mediator for controlling the balance between sphingolipid-induced autophagy and cell death. Moreover, macrophages infected with *L. pneumophila* show an overall reduction of bioactive sphingolipids. Macrophages infected with an *Lp*SPL mutant strain show increased levels of sphingosine compared to wildtype *L. pneumophila* and indeed, *Lp*SPL was confirmed to restrain autophagy by acting on autophagosome biogenesis. Thus, *L. pneumophila* actively modulates sphingolipid metabolism to evade the cell autophagic response (Rolando et al., 2016b; Figure 2).

Another pathogen indicated to manipulate autophagy via sphingolipids is *Salmonella enterica*, an intestinal pathogen that represents a major public health threat due to increasing antibiotic resistance. Similar to *Legionella*, *Salmonella* forms a vacuole, called the *Salmonella-*containing vacuole (SCV). A recent publication extensively reviews the importance of sphingolipids for *Salmonella* infection from adherence to clearance, discussing the interaction of *Salmonella*, sphingolipids and the autophagic machinery (Huang, 2017).

*Salmonella* has been shown to activate the focal adhesion kinase and to recruit it to the SCV. This kinase promotes the activation of the Akt pathway and consequently mTOR is stimulated. This results in the suppression of autophagy and bacterial survival (Owen et al., 2014). More recent studies demonstrate that *Salmonella* does not only inhibit but actively regulates the autophagic pathway. In the early stages of infection, *Salmonella* triggers the amino acid starvation and mTOR inhibition, resulting in the induction of autophagy (Tattoli et al., 2012). However, in later stages the increase of cytosolic amino acid levels in infected cells reactivates mTOR at the surface of the SCV (Narayanan and Edelmann, 2014; Figure 2).

Inhibition of Akt signaling as well as the activation of extracellular signal-regulated kinase (ERK) 1/2 activity is associated with autophagy in colon adenocarcinoma cells (e.g., HT29 and HCT-15) (Ogier-Denis et al., 2000; Kanazawa et al., 2004; Ellington et al., 2006). In this regard, activated ERK was shown to upregulate beclin-1 expression, resulting in the induction of autophagy (Liu et al., 2012). Inhibition of the *de novo* biosynthesis of sphingolipids by myriocin, an inhibitor of the enzyme serine palmitoyl transferase, leads to decreased autophagy through the activation of Akt and downregulation of beclin-1 (Scarlatti et al., 2004). Moreover, myriocin downregulates ERK and represses the membrane recruitment of NOD2 and ATG16L1. The interaction of ATG16L1 and NOD2 in epithelial cells leads to autophagic degradation of *Salmonella*. In addition, myriocin decreased the *Salmonella*-induced LC3-II expression (Huang, 2016). These findings suggest that sphingolipids may play a role in *Salmonella*-induced cellular autophagy of damaged SCVs.

The cell ubiquitinates SCVs to trigger nuclear factor-κB (NF-κB) activation, which can lead to a reduction of bacterial propagation by the induction of inflammation. Furthermore, *Salmonella* escaping to the cytosol are tagged by a dense polyubiquitin coat (Narayanan and Edelmann, 2014). Both ceramide and NSM2 are connected to the regulation or modulation of protein ubiquitination and subsequent degradation (Chapman et al., 2005; Dobierzewska et al., 2011). Taking these facts into consideration, it is possible that sphingolipids play a role in the ubiquitination of the SCV.

## Better Safe Than Sorry - Sphingolipids as Antimicrobials

Sphingolipids and sphingolipid signaling contribute to the immune response upon bacterial infection. Acid sphingomyelinase and ceramide play a decisive role in bacterial internalization and inflammatory response (Li et al., 2019). Sphingolipids, however, can also be applied as antimicrobial substances, regulating the growth and propagation of bacteria.

Around 30 years ago Bibel and colleagues pioneered the investigation of antimicrobial effects of sphingolipids. They reported that sphinganine influences the growth of *N. meningitidis* and *Acinetobacter lwoffii* and damages the cell wall of *S. aureus* (Bibel et al., 1993). Moreover, they tested sphinganine on human volunteers as a preventative antiseptic against subsequently applied *S. aureus* and observed an up to three-log reduction in the population of target micro-organisms compared to untreated controls (Bibel et al., 1995). Furthermore, it was shown that sphingosine effectively killed *S. aureus*, *Streptococcus pyogenes*, *Micrococcus luteus*, *Propionibacterium acnes*, *Staphylococcus epidermidis* and moderately killed *P. aeruginosa*. However, sphingosine was demonstrated to not influence the growth of *E. coli* and *Serratia marcescens* (Bibel et al., 1992). Although more research is needed to understand these differences in the effect of sphingosine, one can speculate that it might be related to the fact that *E. coli* and *S. marcescens* are Gram-negative enterobacteria, as opposed to the others, which are Gram-positive.

The increasing lack of antibiotic treatments due to the development of resistances demands novel approaches for therapies. A better understanding of the antibacterial effect of sphingolipids may offer novel targets for treatment. Within the last decade, the number of publications reporting antimicrobial effects of different derivatives of sphingolipids has increased.

For instance, Banhart et al. (2014) demonstrated that the sphingomyelin synthase inhibitor D609, which has an effect on the uptake of fluorescently labeled ceramide by *Chlamydia muridarum* (Elwell et al., 2011), reduces the propagation of *C. trachomatis*. To better understand the impact of sphingomyelin production on the growth of *C. trachomatis*, they synthesized several ceramide derivatives, such as nitrobenzooxadiazole (NBD)-labeled 1-*O*-methyl-ceramide-C_16_. This derivative resembles to a large extent a compound called 1-*O*-methyl-C_6_-NBD-ceramide, which has been shown not to be converted to sphingomyelin. Interestingly, the treatment with this newly synthetized ceramide inhibits chlamydial growth similar to chloramphenicol and 17 times more effectively than D609 (Banhart et al., 2014).

In another study, the antibacterial activity of sphingosine, as well as short-chain C6 and long-chain C16-ceramides and azido functionalized ceramide analogs was tested. The study revealed that short-chain ceramides and a ω-azido-C6-ceramide had antibacterial effects on *N. meningitidis* and *N. gonorrhoeae*. The uptake of ceramides by *Neisseria* happened rapidly within 5 min, and the killing occurred within 2 h. In contrast to *Neisseria*, these analogs did not display any effects on *E. coli* and *S. aureus* (Becam et al., 2017). However, *E. coli* and *S. aureus* were shown to be efficiently killed by the treatment of dihydrosphingosine and sphingosine (Fischer et al., 2013). During *P. aeruginosa* infection, lower levels of sphingosine were observed due to a reduced activity of acid ceramidase, catalyzing the reaction of ceramide to sphingosine. By normalization of sphingosine levels, the susceptibility to *P. aeruginosa* could be decreased (Pewzner-Jung et al., 2014). Sphingosine, sphinganine and phytosphingosine were demonstrated to have a strong effect on biofilm formation and adherence of *Streptococcus mutans* (Cukkemane et al., 2015; Figure 2).

Beside host sphingolipids or chemically synthesized sphingolipids, extracted sphingolipids of plants have antibacterial activity, as well. For example, the sphingolipids ficusamide, (S)-(−) oxypeucedanin hydrate and (R)-(+) oxypeucedanin hydrate of *Ficus exasperata* showed antibacterial activity. While ficusamide had only a low activity against *E. coli*, (S)-(−) oxypeucedanin hydrate and (R)-(+) oxypeucedanin hydrate showed significant activity against *Bacillus cereus* (Dongfack et al., 2012).

## Concluding Remarks

In the recent years, we have become increasingly aware of the importance of lipids, and sphingolipids in particular, for the processes of infection with and defense against pathogenic bacteria. Bacterial invasion or uptake are often mediated by bacterial attachment to glycosphingolipids or regulated by the increase in plasma membrane ceramide. Bacterial internalization can be a mechanism that increases pathogen survival, but sometimes it is also a part of the immunity, where again sphingolipids participate in the destruction of bacteria through regulating phagosome/lysosome fusion, apoptosis, or the inflammatory response. Intracellular bacteria face the challenges of survival within the cell and here also sphingolipids can be a tool to control autophagy and enable survival, or serve as building blocks for bacterial inclusions, ensuring their reproduction. However, sphingolipids can be applied as antimicrobials, as well, negatively influencing bacterial growth and biofilm formation. Understanding exact mechanisms behind these processes remains a challenge for the future and, in the wake of increasing antibiotic resistance, will be of great value in our fight against bacterial pathogens.

## Author Contributions

TK and VK-P wrote the manuscript and prepared the figures.

## Conflict of Interest

The authors declare that the research was conducted in the absence of any commercial or financial relationships that could be construed as a potential conflict of interest. The reviewer PE declared a past supervisory role with one of the authors TK to the handling Editor.

## References

[B1] Agaisse H. Derre I. (2014). Expression of the effector protein IncD in *Chlamydia trachomatis* mediates recruitment of the lipid transfer protein CERT and the endoplasmic reticulum-resident protein VAPB to the inclusion membrane. *Infect. Immun.* 82 2037–2047. 10.1128/IAI.01530-14 24595143 PMC3993449

[B2] Bajwa A. Rosin D. L. Chroscicki P. Lee S. Dondeti K. Ye H. (2015). Sphingosine 1-phosphate receptor-1 enhances mitochondrial function and reduces cisplatin-induced tubule injury. *J. Am. Soc. Nephrol.* 26 908–925. 10.1681/ASN.2013121351 25145931 PMC4378101

[B3] Banhart S. Saied E. M. Martini A. Koch S. Aeberhard L. Madela K. (2014). Improved plaque assay identifies a novel anti-Chlamydia ceramide derivative with altered intracellular localization. *Antimicrob. Agents Chemother.* 58 5537–5546. 10.1128/AAC.03457-14 25001308 PMC4135853

[B4] Becam J. Walter T. Burgert A. Schlegel J. Sauer M. Seibel J. (2017). Antibacterial activity of ceramide and ceramide analogs against pathogenic *Neisseria*. *Sci. Rep.* 7:17627. 10.1038/s41598-017-18071-w 29247204 PMC5732201

[B5] Becker K. A. Fahsel B. Kemper H. Mayeres J. Li C. Wilker B. (2017). Staphylococcus aureus alpha-toxin disrupts endothelial-cell tight junctions via acid sphingomyelinase and ceramide. *Infect. Immun.* 86:e00606-17. 10.1128/IAI.00606-17 29084896 PMC5736828

[B6] Bibel D. J. Aly R. Shah S. Shinefield H. R. (1993). Sphingosines: antimicrobial barriers of the skin. *Acta Derm. Venereol.* 73 407–411. 7906449 10.2340/0001555573407411

[B7] Bibel D. J. Aly R. Shinefield H. R. (1992). Antimicrobial activity of sphingosines. *J. Invest. Dermatol.* 98 269–273. 1545135 10.1111/1523-1747.ep12497842

[B8] Bibel D. J. Aly R. Shinefield H. R. (1995). Topical sphingolipids in antisepsis and antifungal therapy. *Clin. Exp. Dermatol* 20 395–400. 10.1111/j.1365-2230.1995.tb01356.x 8593716

[B9] Capmany A. Damiani M. T. (2010). Chlamydia trachomatis intercepts Golgi-derived sphingolipids through a Rab14-mediated transport required for bacterial development and replication. *PLoS One* 5:e14084. 10.1371/journal.pone.0014084 21124879 PMC2989924

[B10] Capmany A. Gambarte Tudela J. Alonso Bivou M. Damiani M. T. (2019). Akt/AS160 signaling pathway inhibition impairs infection by decreasing Rab14-controlled sphingolipids delivery to chlamydial inclusions. *Front. Microbiol.* 10:666. 10.3389/fmicb.2019.00666 31001235 PMC6456686

[B11] Castro-Garza J. González-Salazar F. Quinn F. D. Karls R. K. De La Garza-Salinas L. H. Guzmán-de la Garza F. J. (2016). An acidic sphingomyelinase Type C activity from *Mycobacterium tuberculosis*. *Rev. Argent. Microbiol.* 48 21–26. 10.1016/j.ram.2016.01.001 26948102

[B12] Cazalet C. Rusniok C. Bruggemann H. Zidane N. Magnier A. Ma L. (2004). Evidence in the *Legionella pneumophila* genome for exploitation of host cell functions and high genome plasticity. *Nat. Genet.* 36 1165–1173. 10.1038/ng1447 15467720

[B13] Chapman H. Ramstrom C. Korhonen L. Laine M. Wann K. T. Lindholm D. (2005). Downregulation of the HERG (KCNH2) K(+) channel by ceramide: evidence for ubiquitin-mediated lysosomal degradation. *J. Cell Sci.* 118 5325–5334. 10.1242/jcs.02635 16263765

[B14] Cukkemane N. Bikker F. J. Nazmi K. Brand H. S. Sotres J. Lindh L. (2015). Anti-adherence and bactericidal activity of sphingolipids against *Streptococcus mutans*. *Eur. J. Oral Sci.* 123 221–227. 10.1111/eos.12200 26094809

[B15] Cuvillier O. Pirianov G. Kleuser B. Vanek P. G. Coso O. A. Gutkind J. S. (1996). Suppression of ceramide-mediated programmed cell death by sphingosine-1-phosphate. *Nature* 381 800–803. 10.1038/381800a0 8657285

[B16] Davaille J. Gallois C. Habib A. Li L. Mallat A. Tao J. (2000). Antiproliferative properties of sphingosine 1-phosphate in human hepatic myofibroblasts: a cyclooxygenase-2 mediated pathway. *J. Biol. Chem.* 275 34628–34633. 10.1074/jbc.m006393200 10942778

[B17] Deretic V. Saitoh T. Akira S. (2013). Autophagy in infection, inflammation and immunity. *Nat. Rev. Immunol.* 13 722–737. 10.1038/nri3532 24064518 PMC5340150

[B18] Dobierzewska A. Giltiay N. V. Sabapathi S. Karakashian A. A. Nikolova-Karakashian M. N. (2011). Protein phosphatase 2A and neutral sphingomyelinase 2 regulate IRAK-1 protein ubiquitination and degradation in response to interleukin-1beta. *J. Biol. Chem.* 286 32064–32073. 10.1074/jbc.M111.238030 21708940 PMC3173202

[B19] Dongfack M. D. Lallemand M. C. Kuete V. Mbazoa C. D. Wansi J. D. Trinh-van-Dufat H. (2012). A new sphingolipid and furanocoumarins with antimicrobial activity from *Ficus exasperata*. *Chem. Pharm. Bull.* 60 1072–1075. 10.1248/cpb.c12-00279 22863713

[B20] Ebenezer D. L. Berdyshev E. V. Bronova I. A. Liu Y. Tiruppathi C. Komarova Y. (2019). *Pseudomonas aeruginosa* stimulates nuclear sphingosine-1-phosphate generation and epigenetic regulation of lung inflammatory injury. *Thorax* 74 579–591. 10.1136/thoraxjnl-2018-212378 30723184 PMC6834354

[B21] Ellington A. A. Berhow M. A. Singletary K. W. (2006). Inhibition of Akt signaling and enhanced ERK1/2 activity are involved in induction of macroautophagy by triterpenoid B-group soyasaponins in colon cancer cells. *Carcinogenesis* 27 298–306. 10.1093/carcin/bgi214 16113053

[B22] Elwell C. A. Jiang S. Kim J. H. Lee A. Wittmann T. Hanada K. (2011). Chlamydia trachomatis co-opts GBF1 and CERT to acquire host sphingomyelin for distinct roles during intracellular development. *PLoS Pathog.* 7:e1002198. 10.1371/journal.ppat.1002198 21909260 PMC3164637

[B23] Faulstich M. Hagen F. Avota E. Kozjak-Pavlovic V. Winkler A.-C. Xian Y. (2015). Neutral sphingomyelinase 2 is a key factor for PorB-dependent invasion of *Neisseria gonorrhoeae*. *Cell Microbiol.* 17 241–253. 10.1111/cmi.12361 25224994

[B24] Fischer C. L. Walters K. S. Drake D. R. Blanchette D. R. Dawson D. V. Brogden K. A. (2013). Sphingoid bases are taken up by *Escherichia coli* and *Staphylococcus aureus* and induce ultrastructural damage. *Skin Pharmacol. Physiol.* 26 36–44. 10.1159/000343175 23128426 PMC3634627

[B25] Gault C. R. Obeid L. M. Hannun Y. A. (2010). An overview of sphingolipid metabolism: from synthesis to breakdown. *Adv. Exp. Med. Biol.* 688 1–23. 10.1007/978-1-4419-6741-1_1 20919643 PMC3069696

[B26] Gillard B. K. Clement R. G. Marcus D. M. (1998). Variations among cell lines in the synthesis of sphingolipids in de novo and recycling pathways. *Glycobiology* 8 885–890. 10.1093/glycob/8.9.885 9675221

[B27] Gomez-Valero L. Buchrieser C. (2013). Genome dynamics in *Legionella*: the basis of versatility and adaptation to intracellular replication. *Cold Spring Harb. Perspect. Med.* 3:a009993. 10.1101/cshperspect.a009993 23732852 PMC3662349

[B28] Grassmé H. Gulbins E. Brenner B. Ferlinz K. Sandhoff K. Harzer K. (1997). Acidic sphingomyelinase mediates entry of *N. gonorrhoeae* into nonphagocytic cells. *Cell* 91 605–615. 10.1016/s0092-8674(00)80448-1 9393854

[B29] Grassme H. Jendrossek V. Riehle A. von Kurthy G. Berger J. Schwarz H. (2003). Host defense against *Pseudomonas aeruginosa* requires ceramide-rich membrane rafts. *Nat. Med.* 9 322–330. 10.1038/nm823 12563314

[B30] Grösch S. Schiffmann S. Geisslinger G. (2012). Chain length-specific properties of ceramides. *Prog. Lipid Res.* 51 50–62. 10.1016/j.plipres.2011.11.001 22133871

[B31] Hackstadt T. Scidmore M. A. Rockey D. D. (1995). Lipid metabolism in *Chlamydia trachomatis*-infected cells: directed trafficking of Golgi-derived sphingolipids to the chlamydial inclusion. *Proc. Natl. Acad. Sci. U.S.A.* 92 4877–4881. 10.1073/pnas.92.11.4877 7761416 PMC41810

[B32] Hanada K. (2005). Sphingolipids in infectious diseases. *Jpn. J. Infect. Dis.* 58 131–148. 15973004

[B33] Harvald E. B. Olsen A. S. B. Faergeman N. J. (2015). Autophagy in the light of sphingolipid metabolism. *Apoptosis* 20 658–670. 10.1007/s10495-015-1108-2 25682163 PMC4376959

[B34] Harvey H. A. Swords W. E. Apicella M. A. (2001). The mimicry of human glycolipids and glycosphingolipids by the lipooligosaccharides of pathogenic *Neisseria* and *Haemophilus*. *J. Autoimmun.* 16 257–262. 10.1006/jaut.2000.0477 11334490

[B35] Hauck C. R. Grassmé H. Bock J. Jendrossek V. Ferlinz K. Meyer T. F. (2000). Acid sphingomyelinase is involved in CEACAM receptor-mediated phagocytosis of *Neisseria gonorrhoeae*. *FEBS Lett.* 478 260–266. 10.1016/s0014-5793(00)01851-2 10930579

[B36] Hernández-Corbacho M. J. Salama M. F. Canals D. Senkal C. E. Obeid L. M. (2017). Sphingolipids in mitochondria. *Biochim. Biophys. Acta Mol. Cell Biol. Lipids* 1862 56–68. 27697478 10.1016/j.bbalip.2016.09.019 PMC5125891

[B37] Herweg J. A. Pons V. Becher D. Hecker M. Krohne G. Barbier J. (2016). Proteomic analysis of the *Simkania*-containing vacuole: the central role of retrograde transport. *Mol. Microbiol.* 99 151–171. 10.1111/mmi.13222 26374382

[B38] Heuer D. Rejman Lipinski A. Machuy N. Karlas A. Wehrens A. Siedler F. (2009). Chlamydia causes fragmentation of the Golgi compartment to ensure reproduction. *Nature* 457 731–735. 10.1038/nature07578 19060882

[B39] Holmgren J. Lönnroth I. Månsson J. Svennerholm L. (1975). Interaction of cholera toxin and membrane GM1 ganglioside of small intestine. *Proc. Natl. Acad. Sci. U.S.A.* 72 2520–2524. 10.1073/pnas.72.7.2520 1058471 PMC432800

[B40] Huang F. C. (2016). De Novo sphingolipid synthesis is essential for *Salmonella*-induced autophagy and human beta-defensin 2 expression in intestinal epithelial cells. *Gut. Pathog.* 8:5. 10.1186/s13099-016-0088-2 26893616 PMC4758167

[B41] Huang F. C. (2017). The role of sphingolipids on innate immunity to intestinal *Salmonella* Infection. *Int. J. Mol. Sci.* 18:1720. 10.3390/ijms18081720 28783107 PMC5578110

[B42] Huang J. Brumell J. H. (2014). Bacteria-autophagy interplay: a battle for survival. *Nat. Rev. Microbiol.* 12 101–114. 10.1038/nrmicro3160 24384599 PMC7097477

[B43] Hugosson S. Angström J. Olsson B.-M. Bergströmlm J. Fredlund H. Olcen P. (1998). Glycosphingolipid binding specificities of *Neisseria meningitidis* and *Haemophilus influenzae*: detection, isolation, and characterization of a binding-active glycosphingolipid from human oropharyngeal epithelium. *J. Biochem.* 124 1138–1152. 10.1093/oxfordjournals.jbchem.a022232 9832619

[B44] James P. F. Zoeller R. A. (1997). Isolation of animal cell mutants defective in long-chain fatty aldehyde dehydrogenase: sensitivity to fatty aldehydes and Schiff’s base modification of phospholipids: implications for Sjörgen-Larsson syndrome. *J. Biol. Chem.* 272 23532–23539. 10.1074/jbc.272.38.23532 9295289

[B45] Kanazawa T. Taneike I. Akaishi R. Yoshizawa F. Furuya N. Fujimura S. (2004). Amino acids and insulin control autophagic proteolysis through different signaling pathways in relation to mTOR in isolated rat hepatocytes. *J. Biol. Chem.* 279 8452–8459. 10.1074/jbc.m306337200 14610086

[B46] Kavaliauskiene S. Dyve Lingelem A. B. Skotland T. Sandvig K. (2017). Protection against shiga toxins. *Toxins* 9:e44. 10.3390/toxins9020044 PMC5331424 28165371

[B47] Koch-Edelmann S. Banhart S. Saied E. M. Rose L. Aeberhard L. Laue M. (2017). The cellular ceramide transport protein CERT promotes *Chlamydia psittaci* infection and controls bacterial sphingolipid uptake. *Cell Microbiol.* 19:e12752. 10.1111/cmi.12752 28544656

[B48] Kolter T. Sandhoff K. (2005). Principles of lysosomal membrane digestion: stimulation of sphingolipid degradation by sphingolipid activator proteins and anionic lysosomal lipids. *Annu. Rev. Cell Dev. Biol.* 21 81–103. 10.1146/annurev.cellbio.21.122303.120013 16212488

[B49] Kühlewein C. Rechner C. Meyer T. F. Rudel T. (2006). Low-phosphate-dependent invasion resembles a general way for *Neisseria gonorrhoeae* to enter host cells. *Infect. Immun.* 74 4266–4273. 10.1128/iai.00215-06 16790801 PMC1489691

[B50] Lépine S. Allegood J. C. Park M. Dent P. Milstien S. Spiegel S. (2011). Sphingosine-1-phosphate phosphohydrolase-1 regulates ER stress-induced autophagy. *Cell Death Differ.* 18 350–361. 10.1038/cdd.2010.104 20798685 PMC3131882

[B51] Li C. Wang A. Wu Y. Gulbins E. Grassme H. Zhao Z. (2019). Acid sphingomyelinase-ceramide system in bacterial infections. *Cell Physiol. Biochem* 52 280–301. 10.33594/000000021 30816675

[B52] Liu H. Toman R. E. Goparaju S. K. Maceyka M. Nava V. E. Sankala H. (2003). sphingosine kinase type 2 is a putative BH3-only protein that induces apoptosis. *J. Biol. Chem.* 278 40330–40336. 10.1074/jbc.m304455200 12835323

[B53] Liu Y. Yang Y. Ye Y. C. Shi Q. F. Chai K. Tashiro S. (2012). Activation of ERK-p53 and ERK-mediated phosphorylation of Bcl-2 are involved in autophagic cell death induced by the c-Met inhibitor SU11274 in human lung cancer A549 cells. *J. Pharmacol. Sci.* 118 423–432. 10.1254/jphs.11181fp 22466960

[B54] Ma J. Gulbins E. Edwards M. J. Caldwell C. C. Fraunholz M. Becker K. A. (2017). Staphylococcus aureus α-Toxin induces inflammatory cytokines via lysosomal acid sphingomyelinase and ceramides. *Cell Physiol. Biochem.* 43 2170–2184. 10.1159/000484296 29069651

[B55] Managò A. Becker K. A. Carpinteiro A. Wilker B. Soddemann M. Seitz A. P. (2015). *Pseudomonas aeruginosa* pyocyanin induces neutrophil death via mitochondrial reactive oxygen species and mitochondrial acid sphingomyelinase. *Antioxid. Redox. Signal.* 22 1097–1110. 10.1089/ars.2014.5979 25686490 PMC4403017

[B56] Menaldino D. S. Bushnev A. Sun A. Liotta D. C. Symolon H. Desai K. (2003). Sphingoid bases and de novo ceramide synthesis: enzymes involved, pharmacology and mechanisms of action. *Pharmacol. Res.* 47 373–381. 10.1016/s1043-6618(03)00054-9 12676511

[B57] Mencarelli C. Martinez-Martinez P. (2013). Ceramide function in the brain: when a slight tilt is enough. *Cell Mol. Life Sci.* 70 181–203. 10.1007/s00018-012-1038-x 22729185 PMC3535405

[B58] Miller C. Celli J. (2016). Avoidance and subversion of eukaryotic homeostatic autophagy mechanisms by bacterial pathogens. *J. Mol. Biol.* 428 3387–3398. 10.1016/j.jmb.2016.07.007 27456933 PMC5010449

[B59] Mital J. Hackstadt T. (2011). Role for the SRC family kinase Fyn in sphingolipid acquisition by chlamydiae. *Infect. Immun.* 79 4559–4568. 10.1128/IAI.05692-11 21896774 PMC3257913

[B60] Moore E. R. Mead D. J. Dooley C. A. Sager J. Hackstadt T. (2011). The trans-Golgi SNARE syntaxin 6 is recruited to the chlamydial inclusion membrane. *Microbiology* 157 830–838. 10.1099/mic.0.045856-0 21109560 PMC3081085

[B61] Narayanan L. A. Edelmann M. J. (2014). Ubiquitination as an efficient molecular strategy employed in *Salmonella* infection. *Front Immunol* 5:558. 10.3389/fimmu.2014.00558 25505465 PMC4243690

[B62] Newton H. J. Ang D. K. van Driel I. R. Hartland E. L. (2010). Molecular pathogenesis of infections caused by *Legionella pneumophila*. *Clin. Microbiol. Rev.* 23 274–298. 10.1128/CMR.00052-09 20375353 PMC2863363

[B63] Newton J. Lima S. Maceyka M. Spiegel S. (2015). Revisiting the sphingolipid rheostat: evolving concepts in cancer therapy. *Exp. Cell Res.* 333 195–200. 10.1016/j.yexcr.2015.02.025 25770011 PMC4415605

[B64] Ogier-Denis E. Pattingre S. El Benna J. Codogno P. (2000). Erk1/2-dependent phosphorylation of Galpha-interacting protein stimulates its GTPase accelerating activity and autophagy in human colon cancer cells. *J. Biol. Chem.* 275 39090–39095. 10.1074/jbc.m006198200 10993892

[B65] Okino N. Ito M. (2007). Ceramidase enhances phospholipase C-induced hemolysis by *Pseudomonas aeruginosa*. *J. Biol. Chem.* 282 6021–6030. 10.1074/jbc.m603088200 17202150

[B66] Okino N. Tani M. Imayama S. Ito M. (1998). Purification and characterization of a novel ceramidase from *Pseudomonas aeruginosa*. *J. Biol. Chem.* 273 14368–14373. 10.1074/jbc.273.23.14368 9603946

[B67] Owen K. A. Meyer C. B. Bouton A. H. Casanova J. E. (2014). Activation of focal adhesion kinase by *Salmonella* suppresses autophagy via an Akt/mTOR signaling pathway and promotes bacterial survival in macrophages. *PLoS Pathog.* 10:e1004159. 10.1371/journal.ppat.1004159 24901456 PMC4047085

[B68] Peng H. Li C. Kadow S. Henry B. D. Steinmann J. Becker K. A. (2015). Acid sphingomyelinase inhibition protects mice from lung edema and lethal *Staphylococcus aureus* sepsis. *J. Mol. Med.* 93 675–689. 10.1007/s00109-014-1246-y 25616357 PMC4432103

[B69] Perry D. K. (2002). Serine palmitoyltransferase: role in apoptotic de novo ceramide synthesis and other stress responses. *Biochim. Biophys. Acta* 1585 146–152. 10.1016/s1388-1981(02)00335-9 12531548

[B70] Peters S. Schlegel J. Becam J. Avota E. Sauer M. Schubert-Unkmeir A. (2019). *Neisseria meningitidis* type IV pili trigger Ca2+-dependent lysosomal trafficking of the acid sphingomyelinase to enhance surface ceramide levels. *Infect. Immun.* 87:e00410-19. 10.1128/IAI.00410-19 31160362 PMC6652772

[B71] Pewzner-Jung Y. Tavakoli Tabazavareh S. Grassme H. Becker K. A. Japtok L. Steinmann J. (2014). Sphingoid long chain bases prevent lung infection by *Pseudomonas aeruginosa*. *EMBO Mol Med* 6 1205–1214. 10.15252/emmm.201404075 25085879 PMC4197866

[B72] Riboni L. Viani P. Bassi R. Prinetti A. Tettamanti G. (1997). The role of sphingolipids in the process of signal transduction. *Prog. Lipid Res.* 36 153–195. 10.1016/s0163-7827(97)00008-8 9624426

[B73] Rolando M. Escoll P. Buchrieser C. (2016a). *Legionella pneumophila* restrains autophagy by modulating the host’s sphingolipid metabolism. *Autophagy* 12 1053–1054. 10.1080/15548627.2016.1166325 27191778 PMC4922430

[B74] Rolando M. Escoll P. Nora T. Botti J. Boitez V. Bedia C. (2016b). *Legionella pneumophila* S1P-lyase targets host sphingolipid metabolism and restrains autophagy. *Proc. Natl. Acad. Sci. U.S.A.* 113 1901–1906. 10.1073/pnas.1522067113 26831115 PMC4763766

[B75] Rudel T. Kepp O. Kozjak-Pavlovic V. (2010). Interactions between bacterial pathogens and mitochondrial cell death pathways. *Nat. Rev. Microbiol.* 8 693–705. 10.1038/nrmicro2421 20818415

[B76] Saiman L. Prince A. (1993). *Pseudomonas aeruginosa* pili bind to asialoGM1 which is increased on the surface of cystic fibrosis epithelial cells. *J. Clin. Invest.* 92 1875–1880. 10.1172/jci116779 8104958 PMC288352

[B77] Sarkar S. Maceyka M. Hait N. C. Paugh S. W. Sankala H. Milstien S. (2005). Sphingosine kinase 1 is required for migration, proliferation and survival of MCF-7 human breast cancer cells. *FEBS Lett.* 579 5313–5317. 10.1016/j.febslet.2005.08.055 16194537

[B78] Scarlatti F. Bauvy C. Ventruti A. Sala G. Cluzeaud F. Vandewalle A. (2004). Ceramide-mediated macroautophagy involves inhibition of protein kinase B and up-regulation of beclin 1. *J. Biol. Chem.* 279 18384–18391. 10.1074/jbc.m313561200 14970205

[B79] Simonis A. Hebling S. Gulbins E. Schneider-Schaulies S. Schubert-Unkmeir A. (2014). Differential activation of acid sphingomyelinase and ceramide release determines invasiveness of *Neisseria meningitidis* into brain endothelial cells. *PLoS Pathog.* 10:e1004160. 10.1371/journal.ppat.1004160 24945304 PMC4055770

[B80] Simonis A. Schubert-Unkmeir A. (2018). The role of acid sphingomyelinase and modulation of sphingolipid metabolism in bacterial infection. *Biol. Chem.* 399 1135–1146. 10.1515/hsz-2018-0200 29924727

[B81] Simons K. Ikonen E. (1997). Functional rafts in cell membranes. *Nature* 387 569–572. 10.1038/42408 9177342

[B82] Siqueira M. D. S. Ribeiro R. M. Travassos L. H. (2018). Autophagy and its interaction with intracellular bacterial pathogens. *Front. Immunol.* 9:935. 10.3389/fimmu.2018.00935 29875765 PMC5974045

[B83] Speer A. Sun J. Danilchanka O. Meikle V. Rowland J. L. Walter K. (2015). Surface hydrolysis of sphingomyelin by the outer membrane protein Rv0888 supports replication of *Mycobacterium tuberculosis* in macrophages. *Mol. Microbiol.* 97 881–897. 10.1111/mmi.13073 26036301 PMC4671274

[B84] Taniguchi M. Kitatani K. Kondo T. Hashimoto-Nishimura M. Asano S. Hayashi A. (2012). Regulation of autophagy and its associated cell death by “sphingolipid rheostat”: reciprocal role of ceramide and sphingosine 1-phosphate in the mammalian target of rapamycin pathway. *J. Biol. Chem.* 287 39898–39910. 10.1074/jbc.M112.416552 23035115 PMC3501064

[B85] Tattoli I. Sorbara M. T. Vuckovic D. Ling A. Soares F. Carneiro L. A. (2012). Amino acid starvation induced by invasive bacterial pathogens triggers an innate host defense program. *Cell Host Microbe* 11 563–575. 10.1016/j.chom.2012.04.012 22704617

[B86] Tettamanti G. Bassi R. Viani P. Riboni L. (2003). Salvage pathways in glycosphingolipid metabolism. *Biochimie* 85 423–437. 10.1016/s0300-9084(03)00047-6 12770781

[B87] van Ooij C. Kalman L. van I. Nishijima M. Hanada K. Mostov K. (2000). Host cell-derived sphingolipids are required for the intracellular growth of *Chlamydia trachomatis*. *Cell Microbiol.* 2 627–637. 10.1046/j.1462-5822.2000.00077.x 11207614

[B88] Viswanathan G. Jafurulla M. Kumar G. A. Raghunand T. R. Chattopadhyay A. (2018). Macrophage sphingolipids are essential for the entry of mycobacteria. *Chem. Phys. Lipids* 213 25–31. 10.1016/j.chemphyslip.2018.03.004 29526700

[B89] Wolf K. Hackstadt T. (2001). Sphingomyelin trafficking in *Chlamydia pneumoniae*-infected cells. *Cell Microbiol.* 3 145–152. 10.1046/j.1462-5822.2001.00098.x 11260137

[B90] Young M. M. Kester M. Wang H. G. (2013). Sphingolipids: regulators of crosstalk between apoptosis and autophagy. *J. Lipid Res.* 54 5–19. 10.1194/jlr.R031278 23152582 PMC3520539

[B91] Young M. M. Wang H. G. (2018). Sphingolipids as regulators of autophagy and endocytic trafficking. *Adv. Cancer Res.* 140 27–60. 10.1016/bs.acr.2018.04.008 30060813

[B92] Zhang Y. Li X. Carpinteiro A. Gulbins E. (2008). Acid sphingomyelinase amplifies redox signaling in *Pseudomonas aeruginosa*-induced macrophage apoptosis. *J. Immunol.* 181 4247–4254. 10.4049/jimmunol.181.6.4247 18768882

